# T Cell Receptor Repertoires Across the Continuum of Vascular, Myocardial, and Age‐Related Diseases

**DOI:** 10.1111/imr.70125

**Published:** 2026-04-19

**Authors:** Leon Richter, João Dias‐Ferreira, Gustavo Campos Ramos, DiyaaElDin Ashour

**Affiliations:** ^1^ Department of Internal Medicine I University Hospital Würzburg Würzburg Germany; ^2^ Comprehensive Heart Failure Centre University Hospital Würzburg Würzburg Germany; ^3^ Faculty of Sciences of the University of Porto (FCUP) Porto Portugal

**Keywords:** cardiovascular diseases, heart failure, immune aging, myocardial infraction, myocarditis, T cell receptor

## Abstract

Cardiovascular diseases (CVD) are shaped by a complex interplay with immune mechanisms. In particular, the distinct roles of antigen‐specific T cell mechanisms are emerging as critical determinants across a broad spectrum of conditions, ranging from atherosclerosis, myocardial infarction (MI), heart failure (HF), and myocarditis. Because these T cell responses are fundamentally driven by antigen recognition of cardiovascular antigens, understanding T cell clonal dynamics via accessing T cell receptor (TCR) repertoires might provide valuable mechanistic insights for developing targeted diagnostic and therapeutic immunomodulatory approaches in cardiology. In this review, we discuss T cell‐dependent mechanisms and TCR clonal dynamics across various CVD. Moreover, by curating public bulk and single‐cell TCR datasets across different etiologies, we present a first‐in‐class adaptive immune receptor database in cardiovascular diseases (CVD‐TCR database). The discussions and resources herein presented seek to promote an integrated understanding of tissue‐specific immune mechanisms across different CVD, facilitate the identification of shared clonotypes and motifs, and provide a framework for computational modeling of TCR repertoires across the CVD spectrum.

## 1 The Evolving Role of Immune Responses in Cardiovascular Diseases

Cardiovascular diseases (CVD) remain the leading cause of disability‐adjusted life years (DALYs) and deaths worldwide, causing approximately 19 million deaths in 2023 [1]. Among the various pathologies summoned under the CVD umbrella, the ischemic spectrum comprising atherosclerosis, coronary artery disease, and ensuing myocardial infarction accounts for the most common cause of death worldwide [2]. These diseases have been primarily recognized as consequences of vascular lipid accumulation and hemodynamic‐driven pathologies, but in the era of lipid‐lowering therapies and rapid reperfusion, residual inflammation has emerged as a key player driving atherosclerosis and acute coronary syndromes [3], and anti‐inflammatory drugs have started being recommended for cardiovascular prevention [4, 5]. A common consequence of atherosclerosis is myocardial infarction (MI), defined as cardiomyocyte cell death in a setting of ischemia [2]. The acute tissue injury seen in this condition triggers a local inflammatory response that is required for tissue repair but that can also induce further collateral damage [6], ultimately contributing to long‐term ischemic heart failure (HF) [7].

Beyond shaping the ischemic spectrum, the cardio‐immune crosstalk has been proven relevant in a plethora of contexts, including HF associated to pressure overload [8, 9], metabolic syndrome [10], hypertension [11], Chagas' disease [12], and even in genetic cardiomyopathies [13]. Moreover, uncontrolled immune responses directed to cardiac antigens can drive T cell driven myocarditis [14, 15, 16] associated with immune checkpoint inhibition [17] or following infection with cardiotropic viruses [18, 19]. These emerging observations reveal the untapped potential for the development of immunomodulatory interventions in a broad range of CVD.

### 1.1 Immune Surveillance in Healthy Cardiovascular Tissues

The immune system plays key roles in maintaining homeostasis and in pathology development in the cardiovascular niches [20, 21, 22, 23, 24, 25, 26]. Resident embryonic yolk‐sac derived macrophages preferentially localize adjacent to the coronary vasculature, facilitating the remodeling of the primitive coronary plexus through IGF‐1 secretion [27]. Specific subsets of these resident macrophages (LYVE1^+^ MHC‐II^lo^) reside in close proximity to the vascular adventitia and are important in regulating vascular tone and preserving tissue integrity by modulating smooth muscle cell collagen deposition [28, 29]. Beyond resident macrophages, the luminal surface of the endothelium is monitored by Ly‐6C^low^ (in mice) or CD14^dim^ CD16^+^ (in humans) monocytes that patrol the vasculature by crawling along the endothelium [30, 31]. Additionally, the perivascular adipose tissue (PVAT) has been shown to harbor a niche of B1 cells that secrete atheroprotective natural IgM antibodies that help clear oxidation‐specific epitopes [32, 33, 34].

In the healthy myocardium, TIMD4^+^ MHCII^lo^ CCR2^−^ macrophages are seeded during embryogenesis and self‐maintain through local proliferation, independent of the bone marrow‐derived monocyte pool [22, 35]. They play diverse physiological roles including physically coupling with cardiomyocytes via the connexin 43 gap junctions in the atrioventricular node, facilitating electrical conduction [36]. Furthermore, they preserve cardiomyocyte health by engulfing dysfunctional mitochondria ejected by cardiomyocytes, which is required for maintaining their metabolic fitness [37]. The resident cardiac immune cell compartment also includes smaller populations of dendritic cells found in cardiac valves [38], and mast cells located near nerve endings and microvasculature, which have been shown to modulate contractility and vascular tone [39].

### 1.2 From Innate Immune Sensing to Adaptive Immune Responses in Vascular Pathology

The transition from vascular homeostasis to atherosclerosis is a chronic, maladaptive inflammatory process which is initiated in regions of disturbed laminar flow rendering the endothelium susceptible to the retention and subsequent modification of ApoB‐containing lipoproteins in the intima [40, 41]. The oxidative modification of these retained lipids triggers endothelial activation, leading to the upregulation of adhesion molecules such as VCAM‐1 and ICAM‐1 and the release of chemokines such as CCL2 and CX3CL1. This inflammatory signaling leads to the recruitment of CCR2^+^ Ly‐6C^high^ inflammatory monocytes [21, 42, 43]. These monocytes cross the endothelium and differentiate into macrophages which internalize aggregated lipoproteins via scavenger receptors such as SR‐A1 and CD36 which transforms them into foam cells [44, 45]. The intracellular accumulation of lipoproteins leads to the formation of cholesterol crystals that trigger the activation of the NLRP3 inflammasome and the subsequent release of cytokines such as IL‐1β and IL‐6 [46, 47].

As the lesion progresses, dendritic cells present plaque‐derived antigens to T cells. The adaptive response is characterized by a deleterious imbalance: pro‐atherogenic Th1 cells secrete interferon‐γ (IFN‐γ) that activates macrophages and smooth muscle cells, while the protective capacity of regulatory T cells (Tregs) and atheroprotective B1 cells deteriorates [48, 49, 50]. This shifts the immunological burden from innate clearance to antigen‐specific adaptive immune responses that drive chronic lesion progression. Eventually, an exacerbated inflammatory milieu causes macrophages to fail to clear apoptotic cells accumulating in the plaque, which leads to the formation of a necrotic core [51], that can degrade resulting in plaque rupture or erosion and subsequent thrombotic events [52, 53].

### 1.3 Pathological Remodeling in the Myocardial Niche and Progression to Heart Failure

The erosion and rupture of an atherosclerotic plaque can lead to MI, triggering the release of damage‐associated molecular patterns (DAMPs), such as high‐mobility group box 1 (HMGB1), ATP, and mitochondrial DNA [54, 55, 56]. These danger signals initiate a biphasic immunological cascade essential for clearing debris but also capable of inflicting collateral damage if left uncontrolled. The release of chemokines and cytokines such as TNF, IL‐1β, and IL‐6, and CCL2 triggers the mobilization of bone‐marrow‐derived CCR2^+^ monocytes that infiltrate the infarcted myocardium [22, 57, 58, 59]. This influx drives a shift in cardiac macrophage ontogeny, where the TIMD4^+^ resident population of embryonic macrophages gets rapidly replaced by recruited CCR2^+^ monocyte derived‐macrophages [22, 35, 57, 59, 60]. As the inflammatory wave recedes, macrophages switch toward a reparative phenotype [35] that supports the transition of fibroblasts into collagen‐depositing myofibroblasts, which are crucial for stabilizing the ventricular wall [57, 61]. Failure to transition from the inflammatory to reparative phase leads to impaired infarct healing [62, 63], and when the resolution of inflammation is incomplete, or when the heart is subjected to chronic non‐ischemic stress, the immune response drives the progression to HF [64].

The immunological signatures of HF differ fundamentally by etiology. HF with reduced ejection fraction (HFrEF) is often a sequela of MI where persistent cardiomyocyte death and wall stress result in sustained a sterile inflammatory response termed “parainflammation” [65] whereby recruited CCR2^+^ macrophages and activated T cells progressively exacerbate ventricular dilation and cardiac dysfunction [66, 67]. In contrast, HF with preserved ejection fraction (HFpEF) on the other hand is seen as an “outside‐in” mechanism driven by systemic comorbidities such as obesity and hypertension. Systemic inflammation activates the coronary endothelium, leading to the upregulation of adhesion molecules such as VCAM‐1 and the infiltration of immune cells into the perivascular space. HFpEF is driven by diffuse interstitial fibrosis resulting in the stiff, non‐compliant ventricle characteristic of diastolic dysfunction [7, 68, 69]. Regardless of the etiology, the transition to a chronic stage of heart failure is shaped by the recruitment and functional polarization of the adaptive immune system, where the specificity of the T cell response is a key aspect in modulating the pathology.

### 1.4 Cardio‐Immune Crosstalk in the Context of Aging

The efficacy of these homeostatic and reparative immune responses is shaped by the chronological and biological age of the hematopoietic system. Aging is an important aspect impacting both the immune system and the prevalence of various CVDs [70, 71, 72, 73, 74]. While myocarditis predominantly affects young and mid‐aged adults [75, 76], the prevalence of MI and HF dramatically increases with age and in association with comorbidities. In Germany, the average ages of MI are 66 and 75 years old for men and women respectively [77]. These differences in age distribution among patients with different diseases are particularly relevant as far as immunological mechanisms are considered, since the immune system undergoes profound changes with aging too [78, 79].

Immunosenescence reflects a progressive decline in both innate and adaptive immune competence, reducing the overall fitness of the immune system and its ability to respond to new antigens [72, 80]. Age‐induced structural and functional deterioration occurs in multiple organs, including primary and secondary lymphoid tissues such as the thymus and spleen [81, 82]. In the vasculature, senescent immune and stromal cells acquire a senescence‐associated secretory phenotype (SASP), characterized by the release of pro‐inflammatory cytokines, chemokines, and matrix metalloproteinases [83, 84, 85].

An interconnected concept of immune‐aging is inflammaging which refers to a chronic, low‐grade, maladaptive systemic inflammatory state [78], marked by elevated circulating inflammatory markers, including IL‐1, IL‐6, IL‐8, TNF, and CRP [86]. This state is driven in part by the accumulation of senescent cells across tissues, which adopt a senescence‐associated secretory phenotype (SASP) rich in pro‐inflammatory mediators like IL‐6, IL‐8, and TNF [87, 88]. Persistent SASP modifies tissue microenvironments, propagates cellular senescence in neighboring cells, and exacerbates immune dysfunction [89, 90].

Immunosenescence and inflammaging reinforce each other, creating a feedback loop that destabilizes immune homeostasis [91]. Consequences include increased infection risk [92], reduced vaccine efficacy [93], impaired wound healing [94], and worsened outcomes after myocardial injury [95].

The convergence of vascular, myocardial, and aging‐related immunological mechanisms establishes that inflammation is not merely a response to cardiovascular injury, but a primary driver of disease pathology. Chronic immune responses in cardiovascular pathologies are characterized by a persistent antigen‐specific adaptive immune response. In the next section of the review, we will focus on T cell responses in CVDs, their antigenic determinants and their functional polarization toward either pro‐reparative or maladaptive phenotypes depending on the disease progression context.

## 2 T Cell Responses in the Vascular, Myocardial, and Age‐Related Pathologies Continuum

### 2.1 T Cells in Atherosclerosis

Atherosclerosis underlies many CVDs, as rupture of plaques or erosion of arterial walls can lead to sudden vessel occlusion and infarction of myocardial tissue. Oxidative modification of ApoB‐containing LDL generates fragmented and chemically altered forms of ApoB, considered major self‐antigens in atherosclerosis [96, 97, 98]. The autoimmune component of atherosclerosis was initially recognized by identifying circulating autoantibodies targeting LDL [99, 100]. Subsequent studies in mice and humans have demonstrated self‐reactive oxLDL‐ and ApoB‐specific CD4^+^ T cells [101, 102, 103, 104, 105, 106] that support isotype switching and high‐affinity antibody production [99] or CD8^+^ T cell cytotoxicity [96, 107], though their antigen specificity is less studied than CD4^+^ T cells. Additional candidate atherosclerosis autoantigens include heat shock protein 60 [108], β2‐glycoprotein I [109], malondialdehyde‐modified LDL [110, 111].

Under physiological conditions, central and peripheral tolerance restrain autoimmune T cell responses, and many Tregs with self‐specific TCRs exert atheroprotective effects by suppressing inflammation [96, 112]. Peripheral tolerance is maintained through a threshold‐based control system where downstream signaling of the TCR after peptide–MHC interaction, namely Signal 1, requires CD28‐mediated co‐stimulation for full activation, namely Signal 2. In the absence of Signal 2, T cells become anergic or functionally unresponsive [113, 114, 115].

However, under inflammatory atherogenic conditions, Treg cells can become functionally plastic or unstable and acquire pro‐inflammatory transcriptomic and phenotypic characteristics of effector T cell subtypes [49, 96, 104, 105, 116, 117]. Plastic Tregs acquire additional Th1‐, Th17‐, or Tfh‐like effector signatures while maintaining FOXP3 expression, whereas unstable Tregs lose FOXP3, adopt a pro‐inflammatory effector‐like phenotype, and become exTregs with impaired suppressive function [49, 116, 117, 118]. Terminally differentiated exTregs have been identified in humans, characterized by the expression of CD16 and CD56 alongside a loss of FOXP3 expression [119]. Similarly, another study reported a comparable gene expression profile in terminally differentiated TEMRA cells, poised with a cytotoxic gene expression signature [120]. These findings suggest that pathogenic self‐reactive T cells may evolve from formerly atheroprotective Tregs [104]. Drivers of Treg destabilization include chronic antigen exposure, inflammatory signaling, and metabolic reprogramming in inflamed tissues [121, 122, 123]. As tolerance deteriorates, self‐reactive naïve T cells can become activated and clonally expand into pro‐inflammatory or cytotoxic effector T cells [96, 97]. Atherosclerosis is associated with systemic loss of peripheral T cell tolerance, marked by defective expression of immune checkpoint molecules such as CTLA‐4 and PD‐1, clonal expansion of CD4^+^ and CD8^+^ effector T cells, T cell exhaustion, Treg instability and conversion toward a Th17 phenotype, and dysfunctional antigen presentation [97, 120, 124, 125].

In addition to CD4^+^ T helper subsets, CD8^+^ T cells are emerging as important mediators of atherosclerotic disease. Cytotoxic CD8^+^ T cells are present in healthy aortas but become enriched and clonally expanded in atherosclerotic plaques, suggesting antigen‐specific activation [126, 127, 128, 129]. Proposed antigens of plaque‐infiltrating CD8^+^ T cells include HSP60 [108] and ApoB [130, 131], although overall antigen specificity remains poorly defined. Moreover, CD8^+^ T cells specific for viral epitopes (influenza, EBV, CMV, SARS‐CoV‐2) appear within plaques, likely due to cross‐reactivity with self‐antigens through molecular mimicry [132, 133, 134].

CD8^+^ T cell function in atherosclerosis can be double‐edged and stage‐dependent. Proatherogenic effects include the secretion of pro‐inflammatory mediators like interferon‐γ and cytotoxic activity toward lesion‐stabilizing cells, such as vascular smooth muscle cells, via perforin and granzyme B, leading to cell apoptosis, induction of monopoiesis, and lesion destabilization [135, 136, 137]. Consequently, antibody‐mediated CD8^+^ T cell depletion was shown to prevent atherosclerosis in atherosclerosis‐prone mice [135, 136, 137].

Conversely, in established atherosclerosis, CD8^+^ T cell depletion can exacerbate disease [137, 138], and vaccination studies with HSP60 and ApoB indicate that CD8^+^ T cells may also mediate protection and lesion stabilization [108, 130, 131] by eliminating antigen‐presenting cells and macrophages or suppressing CD4^+^ Th17 conversion [138, 139], although these mechanisms are less well characterized than their proatherogenic roles.

Aging, a key risk factor for atherosclerosis, modulates CD8^+^ T cell function [140, 141]. In aged mice, but not in young mice, CD8^+^ T cell depletion was found to attenuate atherogenesis, coinciding with the age‐related accumulation of granzyme K^+^ effector‐memory CD8^+^ T cells within plaques. Adoptive transfer of CD8^+^ T cells from aged wild‐type mice enhanced atherosclerosis in CD8‐depleted recipients, demonstrating that CD8^+^ T cells are both necessary and sufficient for age‐associated atherogenesis [142].

### 2.2 T Cell Roles in Cardiac Injury

Atherosclerotic plaque rupture or erosion can cause acute coronary vessel occlusion and MI. Tissue injury elicits a rapid and complex inflammatory response involving innate immune and adaptive cells [21], with antigen‐specific T cells playing context‐dependent roles in either protecting or exacerbating myocardial damage [143, 144, 145].

Heart‐specific T cells have been identified in patients and experimental models of MI [145, 146, 147, 148, 149, 150, 151, 152, 153], but their relevance extends to pressure overload‐induced heart failure [66, 154, 155, 156, 157] and aging [73, 158]. Chronic T cell activation is consistently linked to ischemic and nonischemic HF progression [66, 154, 155, 156, 157, 159]. In chronic post‐MI stages, proinflammatory, IL‐17, TNF, and IFN‐γ secreting T cells with downregulated FOXP3 expression expand and promote fibrosis and adverse remodeling [148, 159]. Similarly, pressure overload induces persistent IFN‐γ^+^ T cell responses that drive fibroblast to myofibroblast conversion and contribute to fibrosis and pathological remodeling [66, 154, 155, 156, 157]. Although TCR repertoire analyses suggest antigen‐driven T cell expansion [157], the precise cardiac antigens in chronic settings remain unclear.

In contrast, during acute injury, controlled inflammation is essential for debris clearance and proper scar formation [160]. Regulatory FOXP3^+^ CD4^+^ T cells fine‐tune this response and promote tissue repair by limiting monocyte/macrophage recruitment, shaping pro‐healing phenotypes, and restraining fibroblast to myofibroblast conversion [147, 149, 153, 161, 162]. Notably, T cells have been shown to adopt this pro‐healing Treg phenotype upon being activated by cardiac myosin heavy chain alpha (MYHCA) autoantigens, which become exposed upon cardiomyocyte death [149]. Multiple studies support the concept that MI triggers clonal T cell responses to cardiac self‐antigens [163, 164].

Heart‐specific autoimmunity can not only follow cardiac injury but also initiate cardiac damage, as observed in myocarditis. Immunization of susceptible mice with cardiac myosin induces CD4+ T cell‐dependent myocarditis [14, 15, 16, 165, 166, 167], while MYHCA‐specific TCR‐transgenic mice demonstrate that myosin‐reactive T cells can trigger spontaneous myocardial inflammation and drive disease progression toward dilated cardiomyopathy through IFN‐γ and IL‐17 secretion [16, 167]. More recently, MYHCA‐specific CD8^+^ T cells have been implicated in immune checkpoint inhibition‐associated myocarditis [17, 168, 169]. MYHCA has been proposed as a dominant cardiac antigen that T cells respond to in cardiac pathologies [170]. One proposed explanation is that it is not centrally tolerated during thymic selection [171]. On the other hand, other cardiac epitopes have been implicated, including β‐1 adrenergic receptor [172], cardiac troponin [173], and mitochondria‐derived proteins [174, 175]. Beyond cardiac antigen driven T cell responses, chronic viral infections, especially CMV, has been shown to shape the CD8^+^ T cell pool and drive immunosenescence which has been associated with decreased left ventricular function and increased mortality [176, 177]. Additionally, latent CMV infection has been shown to correlate with infarct size and impaired ejection fraction in STEMI patients [178]. Overall, T cell functions in the heart are highly versatile and depend critically on timing and the context of myocardial injury, which has been reviewed in greater detail recently [179].

### 2.3 The Interplay Between Aging and Immunosenescence in Cardiovascular Diseases

The impact of aging on the immune system is diverse and encompasses all different immune cell subsets. For T cells, thymic involution is a central feature that drives their imbalance, resulting in a markedly reduced output of naïve T cells with age [180]. Concurrently, the number and proportion of memory T cells increase due to impaired peripheral maintenance of naïve T cells through homeostatic proliferation and acquisition of a memory‐like phenotype [181, 182, 183]. Lifelong antigenic exposure, for example to chronic viral infections, further drives expansion of the memory T cell compartment at the expense of the naïve pool, accompanied by a pronounced reduction in TCR repertoire diversity [184, 185, 186]. This persistent antigenic stimulation also promotes the accumulation of dysfunctional, terminally differentiated, yet pro‐inflammatory T cells [187, 188]. These senescent T cells are characterized by downregulation of co‐stimulatory molecules CD27/CD28 and expression of terminal differentiation markers such as CD57 and KLRG1 [187, 189]. Chronic viral infections, particularly CMV, exacerbate these changes by inducing memory inflation, promoting cellular senescence, and sustaining low‐grade inflammation [190, 191, 192]. As a result, elderly individuals exhibit diminished immune responses and altered susceptibility to autoimmune disease [193, 194].

Extensive evidence connects immune aging to CVD. Pro‐inflammatory cytokines secreted by senescent T cells and macrophages [195, 196] contribute to endothelial dysfunction, impaired vascular remodeling [197], and atherogenesis [198]. Accumulation of senescent cytotoxic CD8^+^CD28^−^ T cells has been associated with vascular dysfunction [199]. In acute MI, senescent CD8^+^CD57^+^ T cells, poised with a pro‐inflammatory and tissue‐homing phenotype, are abundant in the circulation and correlate with cardiovascular mortality [200]. Similarly, IFN‐γ producing CD4^+^CD28^−^ T cells are expanded in patients with unstable angina relative to stable angina [201].

Computational methods that integrate aging induced immune alterations into a unified score [202, 203] also identify increased proportions of immunosenescence‐linked T cell subsets such as CD57^+^ or CD28^−^ CD8^+^ T cells and reduced naïve T cell pools as contributors to accelerated immune aging and heightened cardiovascular risk [202].

One key contributor to immunosenescence‐ and inflammaging‐induced immune alterations is chronic viral antigen exposure, especially to CMV, which profoundly shapes the T cell compartment [186, 204, 205, 206], occupying more than 20% of the circulating CD4^+^ and/or CD8^+^ memory T cell repertoire in some individuals [207]. CMV seroprevalence increases with aging and has been described to reach ≥ 80% in the 8th decade of life [208, 209]. It is linked to increased frequencies of senescent CD4^+^ and CD8^+^ T cells, impaired vascular function, increased aortic stiffness, and higher cardiovascular mortality [177, 210, 211]. Additionally, experimental models indicate that latent CMV infection induces long‐term changes in the cardiac microenvironment, exacerbating inflammation and remodeling after MI [212]. Overall, CMV infection is associated with a substantial increase in CVD incidence [213].

However, even without overt injury or concurrent infection, physiological cardiac aging is associated with increased inflammatory activity. Specifically, IFN‐γ‐secreting effector T cells accumulate in the heart and in heart‐draining mediastinal lymph nodes, influencing myocardial structure and function [73, 214].

TCR specificity is a defining hallmark of T cell responses [215]. While junctional diversity could theoretically generate 10^19^ sequences [216, 217], the functional human αβ TCR repertoire is constrained to ~2.5 × 10^7^–10^8^ unique clonotypes due to the physical constraints of our body [218, 219], biased junctional recombination and thymic selection [220, 221]. This limited effective repertoire is dynamically reshaped by chronic antigen exposure and aging‐associated contraction, contributing to the oligoclonal expansions observed in different chronic inflammatory conditions including CVDs [216].

Understanding how these antigen specific responses are shaped largely relies on technological advancements that allow us to better dissect the highly diverse TCR repertoire, and what specificity a certain TCR carries across different pathologies [222]. Early repertoire studies relied on flow cytometric detection of V chain segments usage by T cells [223], and on CDR3 length variations (a technique called spectratyping/immunoscope) [224]. A skewed V segment usage or CDR3 length distribution indicated an antigen‐driven clonal T cell response. While providing valuable insights, high‐resolution quantitative analysis of the repertoire composition and diversity was only made possible with the advent of next generation sequencing approaches which allowed the sequencing of millions of variable chains simultaneously [221, 225, 226].

**FIGURE 1:**
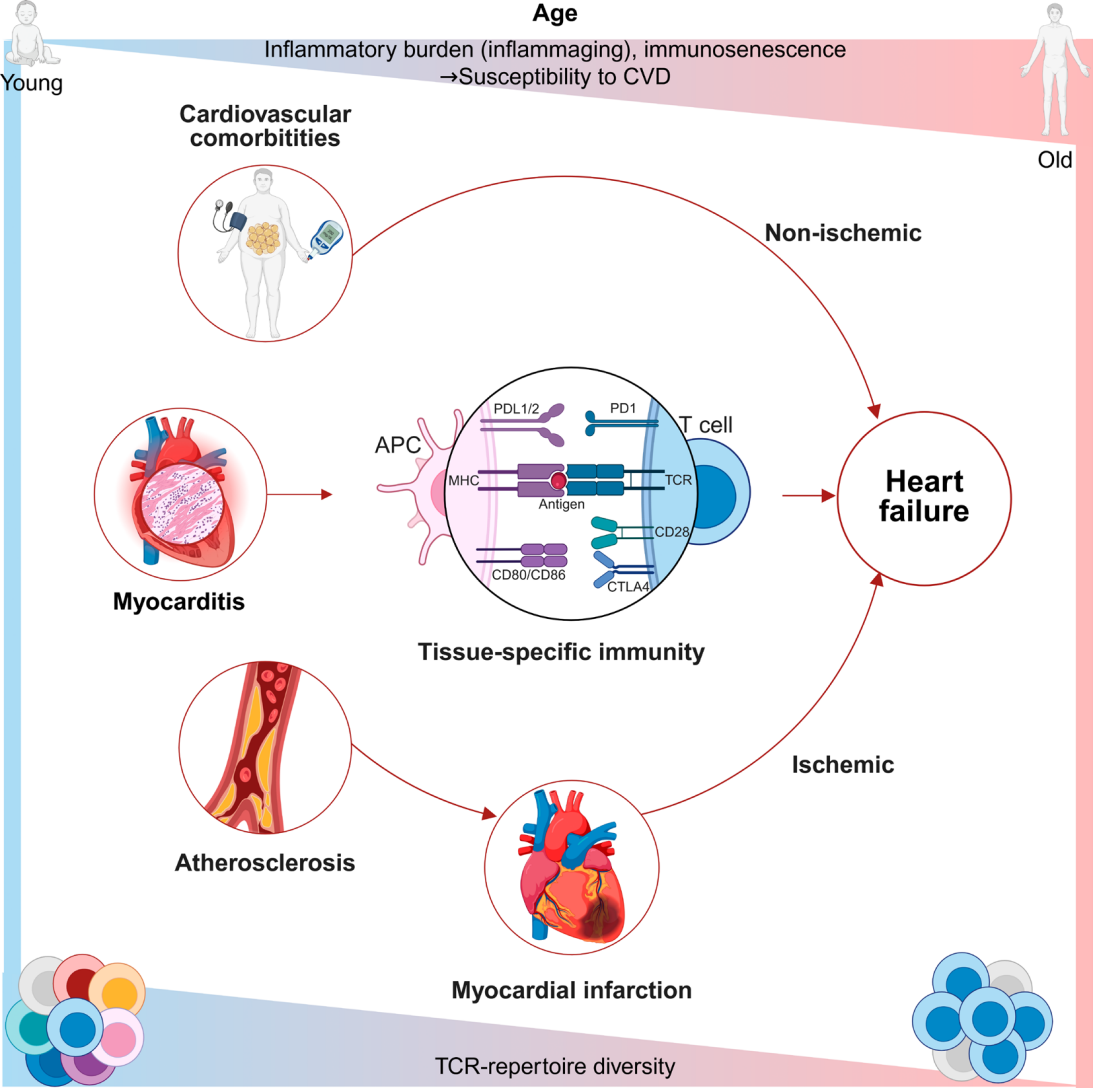
Cardiovascular diseases within the frame of age‐associated immune alterations. Age‐associated immune alterations contribute to CVD. With advancing age, a rising systemic inflammatory burden (“inflammaging”) together with immunosenescence increases overall susceptibility to CVD. These processes disrupt immune homeostasis, shifting the balance between pro‐inflammatory and regulatory cell populations. In the ischemic cardiac disease context, this promotes plaque formation and progression in atherosclerosis, impairs wound healing, and fosters adverse remodeling after myocardial infarction. Cardiovascular comorbidities can also drive a chronic inflammatory milieu within the myocardium that causes non‐ischemic cardiac damage. T cells are key drivers that shape this tissue‐specific immune response and when left uncontrolled can result in heart failure progression. At the same time, reduced TCR‐repertoire diversity reflects a decline in adaptive immune competence, thereby amplifying inflammation and limiting effective tissue repair. Collectively, these age‐related changes within the immune system converge to drive cardiovascular vulnerability and impact disease progression. APC, antigen presenting cell; CD, cluster of differentiation; CTLA4, cytotoxic T‐lymphocyte‐associated protein 4; CVD, cardiovascular disease; MHC, major histocompatibility complex; PD1, programmed cell death protein 1; PDL1/2, programmed death‐ligand 1/2; TCR, T cell receptor.

A major technological block remained, which was to identify the alpha‐beta TCR pairing that would enable cloning and functional characterization of individual antigen specific T cells. Earlier studies utilized hybridoma technology of antigen‐primed T lymphocytes with immortalized thymoma lines to isolate and characterize individual TCR specificities [227]. More recent studies attempted to infer native TCR pairing from bulk sequencing data using combinatorial pooling and statistical co‐occurrence modeling [228], or used single‐cell nested RT‐PCR to physically pair amplicons from sorted populations [229, 230]. High throughput single cell sequencing approaches, including droplet‐based microfluidics [231] and combinatorial fluidic indexing [232], now allow the simultaneous interrogation of T cell phenotypes and paired alpha‐beta TCR identities, which are used in various disease contexts to identify and validate antigen specific T cell responses [17, 233]. Additionally, spatial barcoding of TCR transcripts can now pinpoint local clonal expansion by mapping specific T cells to their tissue microenvironments [234, 235]. We recently reviewed studies that focused on TCR repertoire dynamics in cardiac pathologies [179]. In this review, we broaden this scope to explore studies detailing TCR dynamics in cardiac and vascular pathologies, while incorporating the influence of immune aging and chronic viral infections. We consider these systemic drivers and tissue pathologies to be an inseparable continuum that connects the cardiovascular diseases spectrum rather than separate entities that should be studied in isolation (Figure 1).

## 3 TCR Repertoires in the Vascular, Myocardial, and Age‐Related Pathologies Continuum

### 3.1 TCR Repertoires in Vascular Pathology

Building on the concept that dysregulated T cell immunity contributes to vascular disease, Ma et al. used high‐throughput TCR sequencing on blood samples from patients with essential hypertension and identified markedly altered, less diverse TCR repertoires characterized by clonal expansion and shifts in TRBV/TRBJ gene usage. Notably, reduced diversity and expanded dominant clones independently correlated with carotid intima‐media thickness and subclinical atherosclerosis, particularly in individuals with carotid plaque, indicating that aberrant T cell activation may bridge hypertension and early atherogenesis [236].

Extending this connection between systemic immune dysregulation and local vascular inflammation, several studies have characterized T cell specificity and function within atherosclerotic lesions. Depuydt, Schaftenaar and colleagues profiled T cell clonality in human carotid plaques and paired blood using single‐cell TCR sequencing and demonstrated plaque‐specific clonal expansion of effector CD4^+^ T cells with transcriptional signatures of antigenic stimulation, supporting an autoimmune component in atherosclerosis driven by autoreactive CD4^+^ T cells [120]. To further dissect antigen‐specific mechanisms in atherosclerosis, Wolf et al. interrogated self‐reactive ApoB_978‐993_‐specific CD4^+^ T cells in mice using MHC‐II tetramers. ApoB‐reactive T cells, with preferred TRBV02‐01 and TRBV13‐02 gene usage, displayed a Treg‐like profile in healthy lymph nodes that converted into more clonally expanded‐pathogenic Th1/Th17‐like cells as disease progressed [104, 237]. Consistent with this, another study conducted in the lab of Klaus Ley, employed MHC‐II tetramer‐staining and scRNA‐sequencing on blood samples from HLA‐DRB1*07:01^+^ women with atherosclerosis to demonstrate that ApoB‐specific Tregs shift toward a more memory‐like state, indicating phenotypic instability of the regulatory T cell compartment [105]. Complementing these findings, Roy et al. discovered six novel immunodominant HLA‐II‐restricted ApoB epitopes for humans and, through bulk TCR sequencing, revealed clonal expansion and memory‐linked expression profiles of ApoB‐reactive CD4^+^ T cells. Conserved TCR CDR3 motifs enabled annotation and tracking of ApoB‐specific responses, which correlated with coronary artery disease severity in patients [106, 238].

Freuchet et al. used atherosclerosis‐prone Treg and exTreg lineage‐tracker mice and cross‐species transcriptomic filtering to identify human exTregs as CD3^+^CD4^+^CD16^+^CD56^+^ cytotoxic T cells that are transcriptionally distinct from conventional Tregs. Clonal analysis confirmed that these inflammatory, cytotoxic exTregs arise from Treg clones under atherosclerotic conditions [119].

Beyond CD4^+^ T cell autoreactivity, several studies have examined CD8^+^ T cell involvement. Slütter's group reported enrichment and clonal expansion of activated virus‐specific CD8^+^ T cells within human atherosclerotic lesions as compared to matched blood, yet without detectable viral peptides on HLA‐I molecules, suggesting antigen‐independent activation [134]. Similarly, Chowdhury et al. found clonally expanded T cells exhibiting antigen‐experienced, activated phenotypes in patient plaques. TCR repertoire analysis revealed TCR specificities for viral epitopes homologous to vascular self‐proteins, supporting autoimmune‐like activation through molecular mimicry. Single‐cell transcriptomics indicated that these T cells exhibit proinflammatory, cytolytic, and profibrotic states, implicating them in plaque progression [133].

### 3.2 TCR Repertoires in Myocardial Diseases

Several groups have shown systemic alterations in the circulating TCR repertoire following acute ischemic injury. Using bulk TCR sequencing, these studies show reduced repertoire diversity, elevated clonal expansion, and distinct TRBV/J gene usage in acute coronary syndrome or MI patients compared to individuals with normal coronary arteries. Shared CDR3 sequences across patients further suggested convergent, antigen‐specific T cell responses [239, 240, 241]. To link these repertoire changes to antigen specificity, several studies have analyzed T cells in the infarct‐associated microenvironment. Pedicino et al. showed that epicardial adipose tissue (EAT) in NSTEMI patients displays a pro‐inflammatory proteome and enrichment of CDR3 TRBV21 gene usage not seen in chronic coronary syndrome or mitral valve disease patients. Some patients even shared a specific CDR3 motif potentially recognizing HLA‐A*03:01‐restricted epitopes [242]. Gu et al. performed paired single‐cell RNA/TCR sequencing from coronary thrombi and blood, finding increased clonality in thrombi relative to paired peripheral blood and transcriptional signatures of recent antigen‐specific TCR stimulation. Computational analyses suggested convergent antigen recognition across patients [243].

MYHCA has been identified as a key cardiac antigen in several cardiac pathologies. Rieckmann et al. showed that MYHCA‐specific CD4+ T cells accumulate in the infarcted heart and mediastinal lymph nodes, adopt regulatory phenotypes, and promote cardiac repair in mice. Bulk TCR sequencing showed an oligoclonal T cell response in post‐MI mediastinal lymph node and in cardiac tissue compared to sham [149]. Delgobo et al. furthermore demonstrated that the infarct microenvironment actively drives adoptively transferred MYHCA‐specific CD4^+^ TCR‐M cells [16] toward stable induced Treg phenotypes, comprising two major regulatory lineages enriched for profibrotic/activation markers (e.g., *Tgfb1*) or inhibitory immune checkpoints (e.g., *Pdcd1, Tigit*). These cells suppressed IL‐17 responses and monocyte recruitment, ultimately supporting immune homeostasis and improved post‐MI remodeling. Consistent with this, MHC‐tetramer staining revealed an accumulation of endogenous myosin‐specific T cells in the infarcted heart poised with a regulatory phenotype [153]. Independent of the disease context, Richter et al. further identified and functionally validated TCRs associated with response to a novel MYHCA epitope recognized by murine CD8^+^ T cells, through single‐cell TCR sequencing and reporter assays, thus expanding the toolkit for evaluating myosin‐specific CD8^+^ responses in murine disease on the C57BL/6 genetic background [244].

Apart from MYHCA antigens, our group identified a peptide fragment of the beta1‐adrenergic receptor (ADRB1) that activates CD4^+^ T cells from MI patients carrying HLA‐DRB1*13, providing direct evidence for a defined cardiac autoantigen [172]. Building on this, Rizakou et al. used single‐cell RNA/TCR sequencing of activation‐induced marker (AIM)‐positive cells to identify and functionally validate human TCRs that specifically recognize a novel ADRB1 antigen in the context of HLA‐DRB1*13. The identified T cells were clonally expanded, IFN‐γ‐producing, and showed inter‐patient CDR3 motif sharing. By employing newly validated ADRB1 tetramers, the study furthermore showed that circulating ADRB1‐specific T cells in MI patients predominantly displayed a memory phenotype [245].

Severe immune‐related adverse events like myocarditis can arise from immune checkpoint inhibitor (ICI) anticancer therapy. ICI‐myocarditis provides a contrasting context in which heart‐directed T cell responses become pathogenic. In a *Pdcd1* ^−/‐^ *Ctla4* ^+/−^ mouse model, recapitulating clinical ICI‐myocarditis, Axelrod et al. used paired single‐cell RNA/TCR sequencing on cardiac immune infiltrates and showed that clonally expanding cytotoxic CD8^+^ T cells drive the disease, and that depleting CD8^+^ cells markedly improved survival. Again, MYHCA emerged as the cognate autoantigen in C57BL/6 mice and in patients, with several previously unrecognized immunogenic MYHCA epitopes reported [17]. However, not all ICI‐myocarditis appears MYHCA‐driven. A thorough immune profiling of ICI‐myocarditis patients using single cell RNA/TCR sequencing by Blum et al. found increased cytotoxic CD8^+^ T cells, dendritic cells, and inflammatory fibroblasts in patient hearts as well as distinct shifts in circulating immune cell populations. Yet, expanded TCRs did not recognize classical cardiac antigens such as MYHCA or troponin, indicating alternative antigenic drivers [246].

Myocarditis can also arise from infectious settings, as Vanella et al. report a case of fulminant myocarditis dominated by expanded cytotoxic T cell infiltrates whose TCRs closely matched SARS‐CoV‐2‐specific sequences, indicating viral antigen‐driven cardiac inflammation [247].

Chronic T cell activation following cardiac injury has also been shown to contribute to adverse remodeling and progression to HF. Ischemic failing human hearts were shown to harbor clonally expanded T cells and a highly restricted TCR repertoire dominated by memory and effector CD4^+^ Th1 and cytotoxic CD8^+^ T cells. Notably, patients with common HLA alleles shared T cell clonotypes, suggesting antigen‐driven responses [248]. Mechanistically, it has been shown that myocardial oxidative stress generates isolevuglandin‐modified neoantigens that activate cardiac CD4^+^ T cells. Activated T cells in Nur77‐GFP reporter mice, which express GFP upon TCR engagement, showed skewed TCR repertoires and increased TCR engagement with progressing cardiac dysfunction [157]. In parallel, large‐scale single‐cell RNA/TCR sequencing of cells from human dilated and ischemic cardiomyopathy hearts by Rao et al. revealed substantial infiltration of cytotoxic/exhausted CD8^+^ cells and proinflammatory CD4^+^ cells. Moreover, the study identified a specialized tissue‐resident macrophage subset that interacts with activated endothelial cells, pointing to coordinated inflammatory‐fibrotic cell crosstalk promoting leukocyte infiltration in the failing heart [249].

**FIGURE 2:**
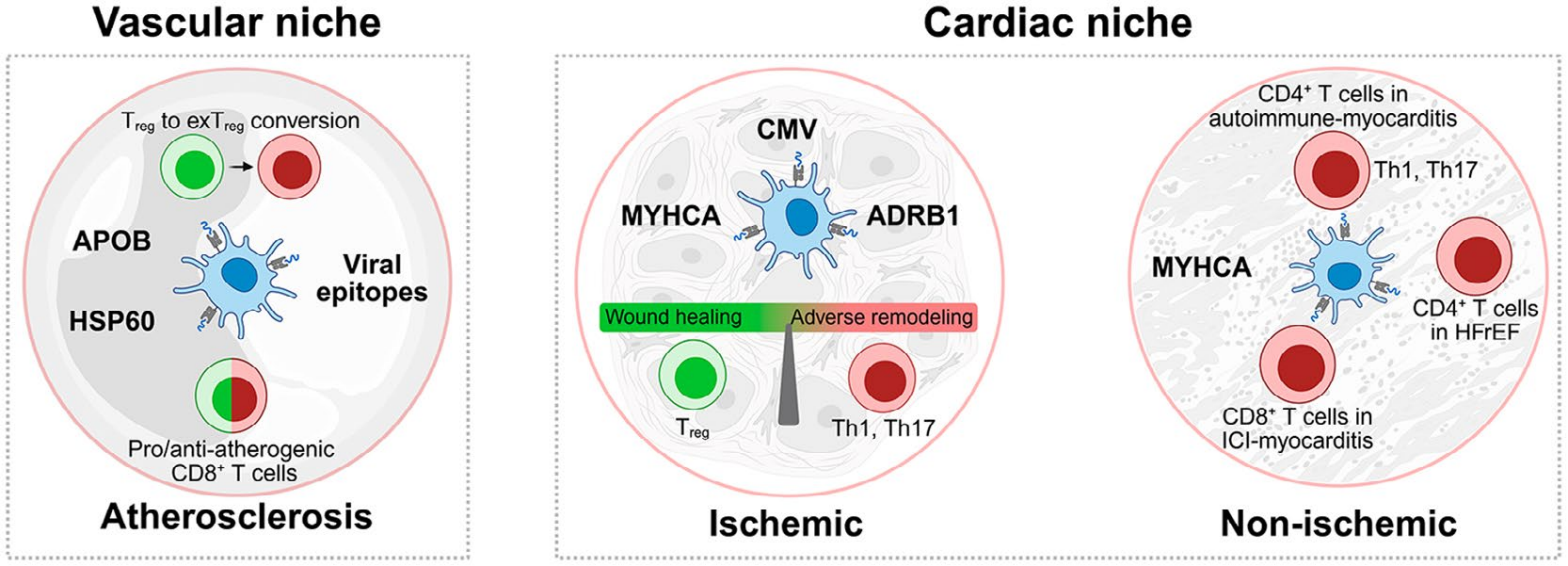
T cell phenotypes and major antigenic determinants in cardiovascular diseases. In atherosclerosis, auto‐reactive Tregs present in healthy subjects at steady‐state conditions undergo pathogenic conversion into a proinflammatory exTreg phenotype as disease progresses. Conversely, acute myocardial injury triggers cardiac specific CD4^+^ T cells that adopt a reparative phenotype. During the chronic stage of both ischemic and non‐ischemic cardiac pathologies, Th1/Th17 effectors promote adverse remodeling and further damage. Likewise, autoimmune myocarditis is characterized by the rapid influx of Th1/Th17 polarized T cells that predominantly respond to myosin antigens and are key initiators of the cardiac pathology. Myosin‐specific CD8^+^ T cells have been shown to infiltrate the myocardium in ICI‐myocarditis and are required to induce cardiac pathology. ADRB1, adrenergic receptor beta 1; ApoB, apolipoprotein B; CD, cluster of differentiation; CMV, cytomegalovirus; HFrEF, heart failure with reduced ejection fraction; HSP60, heat shock protein 60; ICI, immune checkpoint inhibition; MYHCA, myosin heavy chain alpha; Th, T helper cell; Treg, regulatory T cell.

Given its anatomical proximity to the myocardium, epicardial adipose tissue appears to participate in HF‐associated immune activation. Zhang et al. demonstrated that EAT in HF patients harbors clonally expanded, IFN‐γ‐producing effector‐memory T cells with significant TCR clonotype sharing with the adjacent myocardium but not with subcutaneous fat, supporting local antigen‐driven T cell responses [250]. The relevance of EAT extends also to atrial fibrillation (AF), which is a condition contributing to poorer HF outcomes. Single‐cell profiling by Vyas et al. showed that AF is associated with altered CD8^+^ tissue‐resident memory subsets in the EAT of AF patients that localize to the adipose‐atrial interface, where they likely disturb cardiomyocyte calcium flux and promote inflammation and apoptosis [251] (Figure 2).

### 3.3 Aging Impact on TCR Repertoire Dynamics

Aging introduces another layer of immune remodeling that disrupts cardiovascular homeostasis and predisposes to HF. By employing coupled RNA and TCR sequencing on a single cell level, Ashour et al. showed that aged mice exhibit clonal expansion of T cells in the heart and draining lymph nodes, creating an IFN‐γ‐rich inflammatory milieu. Myocardial cell populations, especially cardiomyocytes, displayed increased IFN‐γ response‐signatures and suppressed metabolic pathways, particularly oxidative phosphorylation, mirroring patterns seen in HF and linking immune aging to cardiac vulnerability [214].

In human populations, Terekhova et al. generated a ~2‐million‐cell single‐cell RNA/TCR/BCR dataset from blood of 166 healthy adults aged 25–85. They observed age‐related expansion of multiple CD4^+^ and CD8^+^ effector and memory T cell subsets, along with declines of naïve cells and a previously unrecognized NKG2C^+^GZMB^−^ CD8^+^ subset. These shifts highlight a broad, age‐associated shift in immune homeostasis that likely influences cardiovascular inflammation [252].

### 3.4 Assessing Tissue‐Specific Immune Mechanisms by Leveraging Circulating TCR Repertoires

Immunomodulatory therapy aimed at reducing inflammation in cardiovascular disease is an increasingly promising strategy. While most trials currently focus on modulating the innate immune response, Case et al. used single‐cell transcriptomics and TCR profiling to demonstrate that low‐dose IL‐2 treatment in patients with acute coronary syndrome enrolled in the LILACS trial selectively promotes clonal expansion of effector Treg cells. Longitudinal T cell lineage tracking using distinct TCRs as unique barcodes showed that IL‐2 stabilizes the Treg phenotype, thereby preventing their conversion into effector or memory T cell subsets. Through this mechanism, IL‐2 counteracts the post‐MI inflammatory polarization of the T cell compartment. Notably, CDR3 sequence analysis further revealed that the IL‐2‐expanded Treg clones share antigen specificities associated with atherosclerosis. Whether the IL‐2 expanded Tregs harbor cardiac antigen specificity is yet to be seen. Mechanistically, IL‐2 bypasses BACH2‐mediated repression of the Treg effector program, thereby uncovering a pathway with therapeutic potential not only in cardiovascular disease but also in other immune‐mediated conditions [253, 254].

## 4 Challenges and Approaches to Study TCR Repertoire Dynamics in the CVD Context

As detailed in previous sections, the post‐MI environment is defined by a sterile inflammatory context that harbors a potentially compromised T regulatory cell compartment, due to accumulating vascular inflammation and aging influences. Analyzing the TCR repertoire in this context presents unique challenges compared to classical infection models. Unlike foreign antigens, where high‐affinity clones dominate the response, T cell responses to cardiac injury involve more subtle low‐avidity self‐antigen recognition and potential failure of peripheral tolerance mechanisms governing pre‐existing self‐reactive clones. The healthy T cell repertoire has been shown to harbor self‐reactive precursors that escape thymic deletion [255, 256, 257]. Under homeostatic conditions, these quiescent auto‐reactive clones are effectively constrained by the general population of polyclonal Tregs, which enforce a first tier of bystander suppression [258]. A recent elegant study by Klawon et al. demonstrated that this first tier of regulation fails during active infections coupled with elevated self‐antigen presentation. In such highly inflammatory settings, preventing organ‐specific autoimmunity becomes strictly dependent on a second tier of suppression enforced by antigen‐specific Tregs that share the exact same self‐peptide specificity as the autoreactive T cells [259]. We envision a similar scenario in cardiovascular pathologies, where the expansion of cardiac‐specific T cells may not reflect the de novo priming of a few high‐affinity clones. Rather, the sudden spike of self‐antigens due to cardiac damage within a highly inflammatory milieu leads to a breach in baseline polyclonal tolerance. And tolerance mechanisms become heavily reliant on the presence and function of cardiac‐specific Tregs [149, 153] which become functionally derailed upon chronic inflammatory stimulation [148, 159].

### 4.1 A Curated CVD‐TCR Database

**FIGURE 3:**
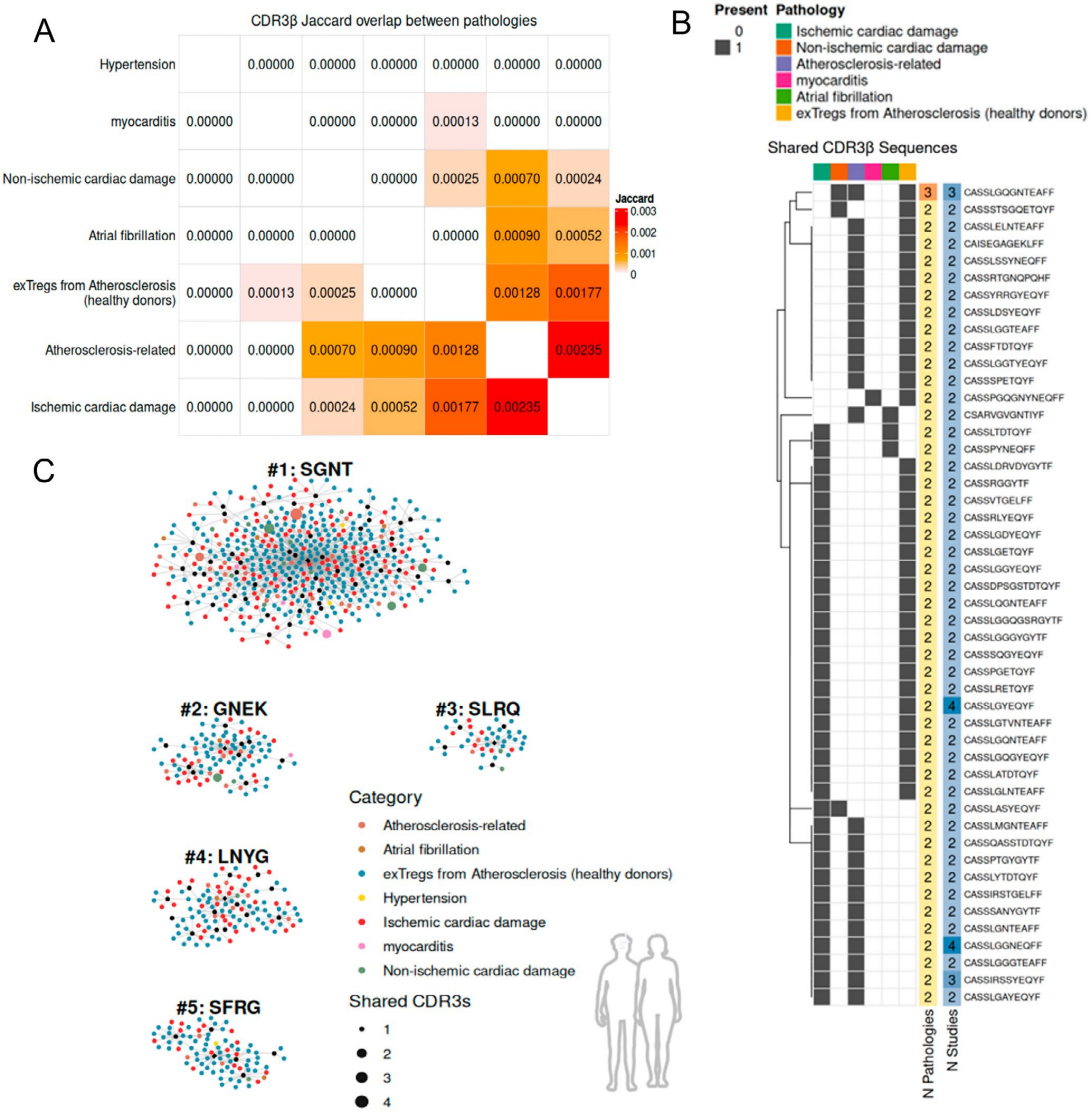
TCRβ repertoire convergence patterns across cardiovascular diseases in human cardiovascular cohorts. Data were aggregated from publicly available bulk and single‐cell TCRβ sequencing studies covering atherosclerosis, MI, HF, myocarditis, hypertension, and atrial fibrillation. Sequences were filtered for clonal expansion (count > 1 for single‐cell; frequency ≥ 10^−4^, and a minimum read count of 5 for bulk TCR data). (A) Pairwise repertoire similarity heatmaps displaying the Jaccard similarity index between unique CDR3β amino acid sequences among distinct cardiovascular pathologies from human data sets. (B) Heatmap showing the distribution of public CDR3β sequences (rows) identified as shared across multiple disease categories (columns). The number of shared pathologies and shared studies per CDR3β are indicated. (C) Network visualization of top expanded TCRβ specificity groups generated using the *turboGliph* implementation of the GLIPH2 algorithm. Human datasets were compared against a reference database composed of 162,165 human TRB CDR3 sequences, obtained from unstimulated healthy subjects [260, 261]. The top 5 networks of core CDR3β motifs that are 4 amino acids in length are shown for human (C) datasets. The networks display central hubs of conserved core motifs that branch into diverse but structurally related motifs (black nodes). Interconnected CDR3β sequences sharing these motifs are colored according to the disease pathology in which the sequence was identified. Since TCR specificity relies on MHC‐restriction, integrative TCR repertoire analysis from patients with different HLA genotypes requires careful interpretation.

**FIGURE 4:**
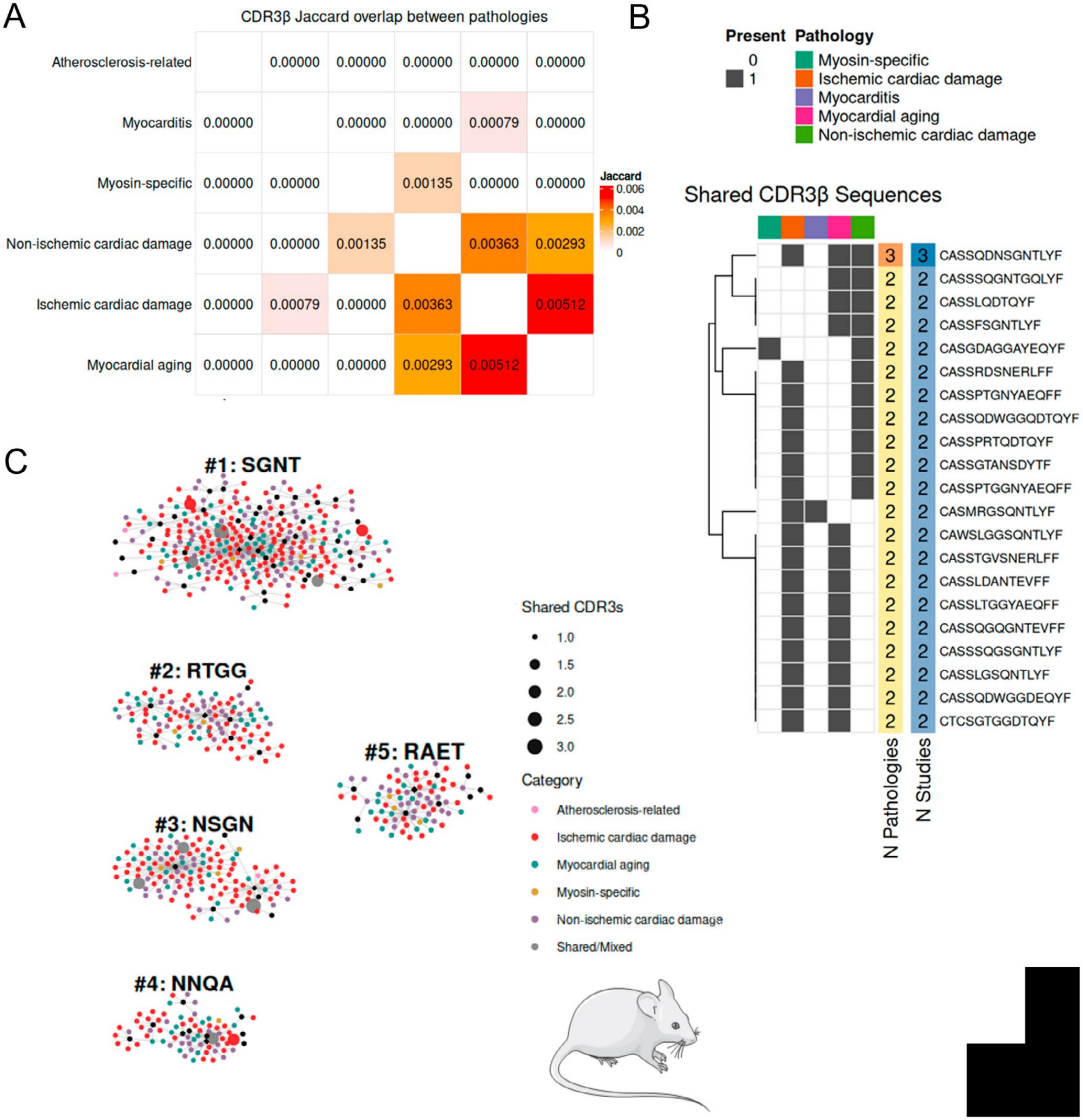
TCRβ repertoire convergence patterns across cardiovascular diseases in murine cardiovascular cohorts. Data were aggregated from publicly available bulk and single‐cell TCRβ sequencing studies covering atherosclerosis, MI, HF, and myocardial aging across different mouse strains. Sequences were filtered for clonal expansion (count > 1 for single‐cell; frequency ≥ 10^−4^, and a minimum read count of 5 for bulk TCR data). (A) Pairwise repertoire similarity heatmaps displaying the Jaccard similarity index between unique CDR3β amino acid sequences among distinct cardiovascular pathologies from murine data sets. (B) Heatmap showing the distribution of public CDR3β sequences (rows) identified as shared across multiple disease categories (columns). The number of shared pathologies and shared studies per CDR3β are indicated. (C) Network visualization of top expanded TCRβ specificity groups generated using the *turboGliph* implementation of the GLIPH2 algorithm. Human and murine datasets were compared against reference databases composed of 78,116 murine TRB CDR3 sequences, obtained from unstimulated healthy subjects [260, 261]. The top 5 networks of core CDR3β motifs that are 4 amino acids in length are shown for mouse datasets. The networks display central hubs of conserved core motifs that branch into diverse but structurally related motifs (black nodes). Interconnected CDR3β sequences sharing these motifs are colored according to the disease pathology in which the sequence was identified. TCR specificity is restricted by MHC; hence, TCR repertoires integrating mouse strains with different genetic backgrounds requires careful interpretation. We herein present a unified mouse CVD‐TCR atlas for the sake of simplicity. However, since the CVD‐TCR database includes MHC restriction information, interested readers can re‐analyze and stratify and TCR motifs by MHC genetics.

As single‐cell and spatial technologies continue to evolve, the challenge in understanding adaptive immune mechanisms in CVDs shifts from identifying expanded T cell clones to interpreting their functional relevance across the cardiovascular continuum. A major analytical challenge in understanding these antigen specific T cell responses in the sterile cardiovascular inflammatory setting is the scarcity of ground‐truth labeled TCR data, which would be crucial to model repertoire dynamics and predict antigen specificity without a priori knowledge of target antigens. Therefore, we compiled and curated various publicly available high‐quality TCR repertoire datasets (listed in Table 1) into a unified CVD‐TCR Database (*GitHub:@RamosImmunoCardiology*) with the aim of determining conserved repertoire patterns across diverse cardiovascular disease etiologies. Our CVD‐TCR Databse was filtered for expanded TCR sequences having a clonal proportion of ≥ 10^−4^, and a minimum read count of 5. for bulk TCR sequencing data or > 1 paired alpha‐beta TCR expanded from single‐cell sequencing data. To assess the global similarity between different disease models, we computed a Jaccard similarity index based on the overlap of unique CDR3β amino acid sequences. For human datasets, TCRs originating from ischemic cardiac damage showed the highest overlap with atherosclerosis‐related TCRs (Figure 3A). While mouse data showed the highest overlap between myocardial aging and ischemic cardiac damage repertoires (Figure 4A). Atherosclerosis‐related mouse TCRs showed no overlap due to the scarcity of publicly available murine atherosclerosis‐annotated TCR data. We additionally screened for exact CDR3β sequence matches, which showed multiple expanded CDR3β sequences shared across different pathologies or reported in different studies within one CVD category, both in human (Figure 3B) and in mouse datasets (Figure 4B).

**TABLE 1:** List of studies performing TCR‐repertoire analysis that were included in TCR‐database curation.

Reference	Species	Pathology	TCR filtration criteria
236	Human	Hypertension	Shortlisted
120	Human	Atherosclerosis	Shortlisted
237	Mouse	Atherosclerosis (healthy donors)	Expanded TCRs > 1
104	Mouse	Atherosclerosis (healthy donors)	Shortlisted
105	Human	Subclinical cardiovascular disease	Expanded TCRs > 1
106, 238	Human	Atherosclerosis (healthy donors)	Shortlisted
119	Human	Atherosclerosis (healthy donors)	Clonal proportion > 10^−4^; min read count = 5
134	Human	Atherosclerosis	Shortlisted
133	Human	Atherosclerosis	Shortlisted
239	Human	Acute myocardial infarction	Shortlisted
240	Human	Acute myocardial infarction, unstable angina	Clonal proportion > 10^−4^; min read count = 5
241	Human	Acute myocardial infarction	Clonal proportion > 10^−4^; min read count = 5
242	Human	Acute myocardial infarction	Shortlisted
243	Human	Acute myocardial infarction	Shortlisted
149	Mouse	Acute myocardial infarction	Clonal proportion > 10^−4^; min read count = 5
16	Mouse	Myocarditis (autoimmune)	NA
153	Mouse	Acute myocardial infarction	Expanded TCRs > 1
244	Mouse	NA	Shortlisted
172	Human	Acute myocardial infarction	Clonal proportion > 10^−4^; min read count = 5
245	Human	Acute myocardial infarction	Shortlisted
17	Mouse, Human	Myocarditis (immune checkpoint inhibition)	Shortlisted
246	Human	Myocarditis (immune checkpoint inhibition)	Shortlisted
247	Human	Myocarditis (viral)	Shortlisted
248	Human	Heart failure (ischemic)	Shortlisted
157	Mouse	Heart failure (nonischemic)	Clonal proportion > 10^−4^; min read count = 5
249	Human	Dilated cardiomyopathy	Expanded TCRs > 1
250	Human	Heart failure	Shortlisted
251	Human	Atrial fibrillation	Expanded TCRs > 1
253	Human	Acute coronary syndrome	Expanded TCRs > 1
214	Mouse	Myocardial aging	Expanded TCRs > 1

Since identical CDR3 sequences are exceedingly rare across individuals due to HLA diversity and the stochastic nature of V(D)J recombination [222], relying solely on exact sequence matches inevitably underestimates the true extent of shared immune responses. A plethora of computational methodologies have emerged that attempt to infer TCR similarity and predict antigen specificity beyond exact sequence matches. These approaches include algorithms that are based on similarity‐weighted Hamming distances or k‐mer matching such as TCRMatch which utilizes a k‐mer based strategy to identify sequence similarities against large reference databases [262], and clustering tools such as GLIPH [260, 261] and TCRdist [263] which allow for clustering diverse TCR sequences into convergent specificity groups based on shared local motifs and global CDR3 similarity. Other tools utilize machine learning and deep learning approaches to extract non‐linear relationships across the repertoire. These tools include protein language models (PLMs) that mathematically encode the physicochemical properties of TCRs to classify disease states from repertoire data without prior knowledge of target antigens [264, 265, 266]. Other models attempt to quantify structural cross‐reactivity by calculating T cell activation scores against mutant epitopes [267] or integrate TCR sequences with single‐cell transcriptomics to categorize T cells simultaneously by receptor identity and functional phenotypes [268, 269]. While being robust, many of these modeling approaches require large scale datasets of known epitope‐specific TCRs for supervised training.

Since our compiled CVD‐TCR Database comprises a heterogeneous mix of bulk and single cell sequencing data with many TCRs lacking annotated cardiac antigens, we instead leveraged GLIPH2 [261] as an unsupervised approach that would allow us to construct convergent TCR‐specificity networks and identify shared repertoire features beyond exact CDR3 sequence matches. While many of the compiled datasets lack HLA information and are thus potentially HLA polymorphic by nature, GLIPH2 prioritizes local CDR3 motif enrichment which often constitutes the primary contact points with the antigenic peptide. This approach can reveal disease‐associated repertoire features even when MHC restriction is not established. GLIPH2 was individually applied to the lists of expanded CDR3β sequences found in the murine and human cohorts. To identify motifs specifically enriched in cardiovascular diseases, the human or murine myocardial data sets were compared with reference databases composed of 162,165 human or 78,116 murine unstimulated TCRβ sequences from repertoires of healthy subjects [260, 261].

This analysis relied on the generation of networks of interconnected CDR3β sequences sharing a common core motif region. It revealed conserved core and diverse but structurally related CDR3β motifs with interconnected CDR3β sequences across different CVD pathologies in humans (Figure 3C) and mouse (Figure 4C). Detailed motif and CDR3β sequences information within each network are shared with the community through the CVD‐TCR Github (@RamosImmunoCardiology), which will be constantly updated as future studies in the field emerge. We expect this compiled atlas of shared and motif‐linked TCRs to grow alongside newly defined CVD‐associated sequences, enabling the community to build robust models defining cardiac‐specific TCR repertoire patterns and to streamline the functional validation of their respective antigens.

## 5 Concluding Remarks

Immuno‐cardiology has gained growing attention in recent years, with new perspectives to leverage immune mechanisms to mitigate the burden of CVDs. In particular, the implication of T cells across a broad range of CVDs has opened new opportunities for understanding tissue‐specific mechanisms. In contrast to innate immune mechanisms, T cell activation depends on the recognition of specific antigen:MHC complexes through their cognate TCR. Therefore, understanding TCR clonal dynamics in CVDs might shed light on key immunological mechanisms that can be exploited for targeted immunomodulation. Moreover, we put forward that the presence of convergent T cell antigen specificities across a broad range of vascular and myocardial diseases might serve as a unifying mechanism integrating different patient groups. The observation of common self‐antigens, such as MYHCA or ApoB antigens, or cross‐reactive viral epitopes suggests a shared adaptive immune continuum that links these pathologies. Ultimately, we envision that the catalogue of cardiovascular TCRs annotated and curated within this work will serve as a key resource to help future studies analyze a growing number of TCR repertoire datasets against this reference repertoire compiled from multiple studies.

## Funding

This work was supported by the German Research foundation (DFG), including the Collaborative Research Centre 1525 “Cardio‐immune interfaces” (grant number 453989101—to G.C.R., UH and SF); the Heisenberg Program (GCR grant number 517001338) and individual grant 411619907. J.D.‐F. was funded by the European Commission under the Erasmus+ program (2024‐1‐PT01‐KA131‐HED‐000214636).

## Conflicts of Interest

The authors declare no conflicts of interest.

## Data Availability

The full catalogue of cardiovascular TCRs will be made available on the Immunocardiology Lab GitHub (GitHub: @RamosImmunoCardiology).
